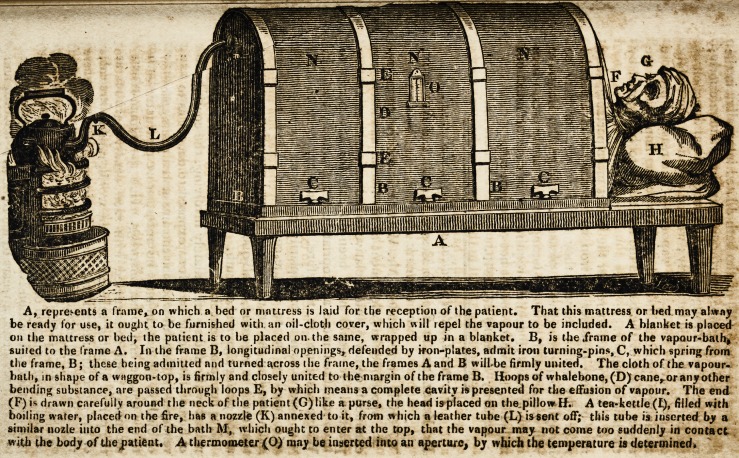# Observations on the Application of Oiled Silk or Oil-Skin, to the Surface of the Human Body

**Published:** 1816-03

**Authors:** Alex. Ramsay

**Affiliations:** Lecturer on Anatomy and Physiology, &c. &c.


THE LONDON

### Medical and Physical Journal.

3 OF VOL. XXXV.] MARCH, 1816. [NO. 205.

"For many fortunate discoveries in medicine, and for the de[illegible]ction of nume-
"rous errors, the world is indebted to the rapid circulation of Monthly
"Journals; and there never existed any work to which the Faculty in
"EUROPE and AMERICA were under deeper obligations than to the
"Medical and Physical Journal of London, now forming a long, but an
"invaluable, series."—RUSH.

*For the London Medical and Physical Journal.*

### *Observations on the Application of Oiled Silk or Oil-Skin, to the Surface of the Human Body;* by ALEX. RAMSAY, M. D. Lecturer on Anatomy and Physiology, &c. &c.

IN the year 1796, I was induced to draw deductions respecting the treatment of the human skin, where the vessels become enfeebled or inert, from the following circumstances:—When I was a student of anatomy, the frequent occurrence of wounds from the scalpel, occasioned my adoption of finger cases formed of oil-skin, which, being impervious to moisture, prevented the danger accruing from the contact of wounded parts with putrid matter. This impervious substance, equally precluded the escape of vapor from the finger, retained the heat, and occasionally the perspiration appeared in a condensed state on the oil-skin. Observing this local influence, I was in the use of recommending to my pupils, in my lectures in Surgeon's-square, in Edinburgh, in 1796, and subsequent years, the application of oil-skin in all cases, where an artificial atmosphere was denoted, in a partial or general manner to the surface.

Whether the universal use of this substance took its rise from those hints I have mentioned, is of little consequence to the public or to me; the proper application of it, however, is of importance to invalids; and, on my late return to Europe, in 1810, I find it hurtful in some cases, from an ignorance of the rationale of its operation. I therefore trust, that a liberal public will forgive my obtruding on their attention this subject, so long familiar to me, and so often productive of the most unexpected happy consequences; nor can this surprise us when we consider the highly vascular and nervous economy of the structure of the skin, a diagram of which I had the pleasure of offering, (No. 184, June 1814,)

1814,) prepared with the cold injection which I have recommended in America, and find now frequent in Europe.* I shall first enumerate a few general circumstances; and then the particular applications of oil-skin.

*General Observations.*—In all cases, the oil-skin ought to be lined with woolly cotton or flannel; this at once prevents the coldness of the silk being perceived when first applied, absorbs perspiration, and has the effect of obviating the chilling sensation experienced after the ceasing of copious perspiration.

I shall be particular, in pointing out, where an outside covering is denoted to prevent friction, because the influence of the substance is lost, whenever the surface is abraided. I should be apt to suppose, that when coarse oiled linen is adopted in place of silk, that the rough side should be exposed to the human skin, as less conduction takes place than from the smooth surface.† The linings, in all cases, ought to be loose, so as to be removed, and dried or washed daily; the surface of the silk ought to be sponged with a slight soap-lather; when the linings are stitched in, and left over night in foot-socks, &c. they become damp in the morning, and partially overthrow the purposes intended.

*Gloves.*—From what has been said, the reader will conclude, that a cotton or flannel glove, distinct from the oil-skin, is to be used, over which the oil-skin glove is to be drawn: when they are separate they can be dried, resume their entire purposes; and, by drawing a common glove over the oil-skin, the heat is increased, and the surface of the silk preserved. The lining and outer glove may be adapted to the state of the patient, in their fabric of cotton or worsted, &c. For children and old people, in gout, rheumatic affections, palsy, &c. these applications seem highly useful.‡

*Waistcoats, &c.* — Waistcoats, or breast-pieces, I have known to banish hæmoptosis, and aleviate asthma: they often excite blisters if a lining is not added; drawers, stockings, and foot socks, are now frequently in use; and local affections of partial organs, as the throat, joints, or surfaces of the skin, &c. are relieved by this application. All these require linings only, as the cloathing and stocking preserve the

---

* See No. 184 of this Journal, alluded to.

† Excepting, however, on this account I must prefer the smooth side toward the skin, as its abraision is thus lessened, and it admits of being more easily sponged and cleaned.

‡ I have known even cutaneous eruptions corrected by this plan of precluding atmospheric influence.

outer outer surface. A complete envelop in a desperate case of dropsy abroad, not only excited sensible perspiration, but occasioned much alleviation of symptoms.

*Bed covers.*—I find, by several experiments on myself, and some on patients, that oil-skin forms a powerful substitute for bed clothes; indeed, a few bed clothes are necessary as interposing substances, to lessen the over accumulation of heat. The sudden condensation of the perspired matter, by the oil-skin in cold weather, reflects the extricated heat so abundantly, as to induce, occasionally, copious perspiration seemingly in a short period. I, therefore, lay the silk over the blankets, with a woolly cotton interposed, as an absorbent, as the oil-skin is usually in a profuse wet state in the morning, and thus injures the blankets. In my own case, I find, that covering from the feet up to the knees sufficiently excites the system; a weighty coverlet spread over the oil-skin, occasions a regular application to the surface of the body, and thus produces a rapid and equal excitement of the external vessels.

*Envelop.*—Where great cold is experienced, or sudden perspiration denoted, would an envelop of flannel, applied to the skin, around which a similar covering of oil-skin may be wrapped, produce the intended effect?—My experiments on myself seem to favour this opinion.*

*Cloak of Oil-skin.*—I have ordered a cloak to be formed of oil-skin, for the purposes of repelling rain or cold, which may be seen at the gentleman's shop named below,† to whom

---

* The improper treatment of domesticated animals, seems to pervert their constitution. Gentlemen of the veterinary art, have observed to me, the frequent failure of sudorific medicines, given to the horse.—Would a covering of oil-skin produce the effect wished, by exciting the cuticular system?—Are not our animals too much confined in houses? I have observed in my travels, that no cattle were so well-conditioned as those that never were housed. Where the constitution is sound, no children, no people suffer so little from inclemency of any kind, as those who do not indulge in warm cloathing. I fear your readers may suspect me as too much bordering on quackery, too sanguine and extended in my proposals and applications of oil-skin, but the unprejudiced man must perceive, that my recommendations flow from the structure of the skin, and my belief of its functions; as also its perversion of structure by too much covering, by debilitating causes, &c. and the necessity of counteracting this state, by a substance adapted to exclude powerful transitions, and to facilitate the operations of nature.

† Mr. John Hargrave, umbrella and parasol manufacturer, and whalebone cutter, No. 29, Bishopsgate-street-within, London.

I have communicated those mechanical notions, not applicable to your readers or the purposes of your Journal.

*Vapour-Bath.*—No circumstance harrowed up my feelings more cruelly than the sufferings of patients in the yellow-fever hospitals, which I visited abroad. The fatigue endured by conveying them from their chambers to the vapor bath, seemed to induce aggravated symptoms.* This occasioned my proposing the plan of the following portable vapour-bath, formed of oiled silk or cloth, or painted cloth. I shall refer to the annexed diagram for conveying a notion of this apparatus.

In yellow fever, in languid cases, in high temperatures of climate, in low typhus, &c. vapour-bath became the only preparatory means by which I quelled the irritation of the stomach, and thus paved the way for medical application.†

I have only to add, that cheapness and durability of an article so extensively useful as the substance, the application of which I have proposed, has become an object of my attention. Mr. John Hargrave has, at his own expence, in a manner equally polite and philanthropic, furnished me with materials, and instituted such experiments as I suggested. This gentleman, therefore, is in possession of my opinions, respecting not only the manner of preparing oiled silk and cloth, but merits my recommending him to public attention and confidence.

---

* In this dreadful malady, so exhausted are the powers of nature occasionally, I have witnessed a patient, in the syncopy of death, from the indiscretion of the nurse permitting him to rise to make water or stool, in place of introducing the bed-pan, and I was forced to permit the unhappy victims to remain longer in the squalid linens they wore than cleanliness seemed to dictate, as shifting them often terminated in fainting, in aggravated symptoms, and even in death.

† In all cases of irritation or spasm, as asthma, colds, incipient fever, &c. vapour-bath seems denoted as a laudable application; and, if the plan proposed here succeeds, much expence incurred by individuals in the use of public baths will be saved, and invalids can always enjoy their benefit at home at an easy rate.—In a future communication, I intend to offer remarks, on the cases where cold-bath, warm-bath, and vapour-bath, are peculiarly denoted—where the above mode of vapour-bath proposed, will be recommended in cases of corpulency, by which means, when assisted by bandaging the arms, legs, and trunk, the vessels acquire habitual tone, and the system may be altered.

A, repre-

Were the nozle (M) of the bath dilated internally, in the shape of a large globe, perforated anteriorly and inferiorly, or a tube passed through the whole longitudinal direction of the bath, perforated inferiorly; would this diffuse more safely and extensively the issuing vapour?—The reason I propose the inferior perforations is that, the top being impervious, the upper surface of the bath is the less liable to be injured by the heated vapour.

If it is required to render the frames more portable, they may be held together by iron in the manner of a parallel ruler, by which means, they fall together, or may be brought to right angles at pleasure. I hope Mr. Hargrave will prepare such baths for letting out for family uses, where common cases of debility require it. In cases of infectious maladies, such baths ought not to be lent out.

*London; January* 27, 1816.

---

*For the London Medical and Physical Journal.*

## Figures and Tables

**Figure f1:**